# Prediction of ground motion and dynamic stress change in Baekdusan (Changbaishan) volcano caused by a North Korean nuclear explosion

**DOI:** 10.1038/srep21477

**Published:** 2016-02-17

**Authors:** Tae-Kyung Hong, Eunseo Choi, Seongjun Park, Jin Soo Shin

**Affiliations:** 1 Yonsei University, Department of Earth System Sciences, 50 Yonsei-ro, Seodaemun-gu Seoul 120-749, South Korea; 2 Center for Earthquake Research and Information, University of Memphis, Memphis, Tennessee 38152, USA; 3 Earthquake Research Center, Korea Institute of Geoscience and Mineral Resources, 92 Gwahang-no, Yuseong-gu, Daejeon 305-350, South Korea

## Abstract

Strong ground motions induce large dynamic stress changes that may disturb the magma chamber of a volcano, thus accelerating the volcanic activity. An underground nuclear explosion test near an active volcano constitutes a direct treat to the volcano. This study examined the dynamic stress changes of the magma chamber of Baekdusan (Changbaishan) that can be induced by hypothetical North Korean nuclear explosions. Seismic waveforms for hypothetical underground nuclear explosions at North Korean test site were calculated by using an empirical Green’s function approach based on a source-spectral model of a nuclear explosion; such a technique is efficient for regions containing poorly constrained velocity structures. The peak ground motions around the volcano were estimated from empirical strong-motion attenuation curves. A hypothetical M7.0 North Korean underground nuclear explosion may produce peak ground accelerations of 0.1684 m/s^2^ in the horizontal direction and 0.0917 m/s^2^ in the vertical direction around the volcano, inducing peak dynamic stress change of 67 kPa on the volcano surface and ~120 kPa in the spherical magma chamber. North Korean underground nuclear explosions with magnitudes of 5.0–7.6 may induce overpressure in the magma chamber of several tens to hundreds of kilopascals.

Baekdusan (Changbaishan) is an active intraplate stratovolcano ~1200 km away from the nearest convergent plate boundary off eastern Japan. The volcano has erupted several times in history, including ~4000 BP, ~2130 BP, 946, 1668, and 1702. The most recent eruption occurred in 1903^1^. The volcanic eruption in ~946 (namely, the Millennium eruption) had a volcanic explosive index (VEI) of 7 and tephra and pyroclastic flow volume of ~100 km^3^; it is one of the largest explosive events in human history^2^,^3^,^4^. The Millennium eruption produced pyroclastic flows of up to 100 m thickness around the caldera and an ash fallout of 4–5 cm in northern Japan^2^,^5^,^6^. Recently, the volcano showed signs of unrest, such as increased seismicity and summit uplift from 2002 to 2005^2^,^7^. Reports have suggested the presence of a high-temperature zone or magma chamber at depths as shallow as 5 km^8^,^9^,^10^, which has increased the concern over the instability of the volcano^11^,^12^.

North Korea has conducted three moderate-sized underground nuclear explosion (UNE) tests in 2006, 2009 and 2013, respectively at a site ~116 km away from Baekdusan (Fig. 1(a)). The magnitudes of the three UNEs are *m*_*b*_ = 4.3, 4.7 and 5.1, respectively^13^,^14^,^15^,^16^ (see, supplementary materials). The North Korean UNEs were well recorded at 109 regional seismic stations in Korea, Japan and China. The regional waveforms and frequency contents varied significantly with the raypaths because of complex crustal structures^17^,^18^,^19^ (Fig. 1(b)). The crustally guided shear waves (*Lg*) and fundamental-mode Rayleigh waves (*Rg*) produce strong ground motions along continental paths^18^,^20^.

Various studies have reported on volcanic eruptions triggered by earthquakes^21^,^22^,^23^,^24^,^25^. There is growing anxiety whether a large future nuclear explosion at the North Korean test site may disturb the magma chamber and cause a volcanic eruption. Baekdusan is located 116 km away from the North Korean test site. It is close enough to be potentially affected by a moderate-sized or large seismic event^26^. The triggering of a volcanic eruption is controlled by various parameters including a stress (pressure) change, the magma composition, the depth and volume of the magma chamber, the viscosity, and local tectonic settings^25^,^27^. The stress (pressure) change plays a crucial role in volcanic eruption.

The internal pressure in a magma chamber can be influenced by a dynamic stress change as well as lithostatic stress field^28^. Previous UNE tests have shown that detonation strength can be greater than M7.0^29^. Large UNEs may produce strong seismic waves over local and regional distances. Dynamic stress changes induced by transient seismic waves can overpressurize the magma and trigger volcanic eruption^30^,^31^,^32^,^33^.

We estimate dynamic stress changes that a hypothetical North Korean UNE can induce in the Baekdusan magma chamber. However, the Korean Peninsula on which Baekdusan is located was formed by a series of continental collision and rifting from the late Permian to mid-Miocene^34^,^35^,^36^, which led to the construction of complex crustal structures^17^,^34^,^37^ (see, supplementary materials). Synthetic calculation of seismic waveforms is not suitable for such regions where the velocity structures are poorly constrained since seismic waveforms are sensitive to the crustal structure and surface topography along the raypath^13^,^19^,^38^. We calculate the seismic ground motions from hypothetical nuclear explosions by using an empirical Green’s function approach based on the seismic waveforms of previous nuclear explosions and a UNE source-spectral model. The peak ground velocities and dynamic pressure changes in the magma chamber of Baekdusan volcano are determined from a numerical model of the dynamic stress coupling and distance-dependent ground motion attenuation.

## Results

### UNE source calibration

A UNE source-spectral model^39^,^40^,^41^ is implemented to calculate the seismic waveforms for a given UNE detonation size. The validity of the UNE source-spectral model is checked, and the apparent seismic moment in the model is calibrated for the UNE magnitude^20^. The spectral-amplitude ratios of seismic records of North Korean UNEs at common stations yield source-spectral amplitude ratios of the UNEs because raypath effects such as geometrical spreading and seismic attenuation are naturally corrected.

**Figure 1:**
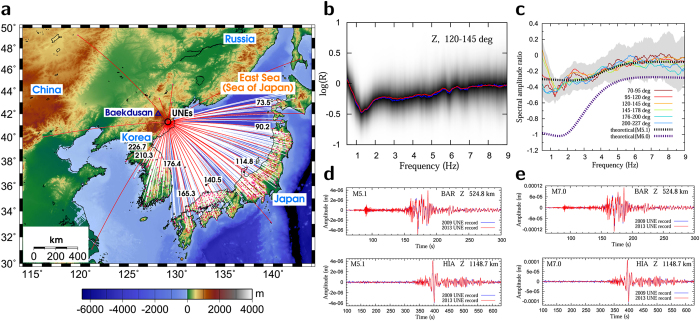
Data and quasi-observed seismic waveform synthesis. (**a**) Map of seismic stations with annotated azimuths, (**b**) stacked spectral-amplitude ratios between the 2009 and 2013 UNE records for stations in the azimuths of 120°–145°, and (**c**) comparison of stacked spectral-amplitude ratios for various azimuthal ranges. The population of the spectral-amplitude ratios is assessed by using a Gaussian distribution function. (**d**) Quasi-observed waveforms at two regional stations (BAR, HIA) for an *m*_*b*_ 5.1 UNE that are calculated based on the 2009 *m*_*b*_ 4.7 UNE. The quasi-observed waveforms match the observed waveforms of the 2013 *m*_*b*_ 5.1 UNE well. (**e**) Comparison between quasi-observed waveforms for *m*_*b*_ 7.0 UNE based on the 2009 and 2013 UNE waveforms. The quasi-observed waveforms match well each other. The map was created by using the software Generic Mapping Tools (http://gmt.soest.hawaii.edu/).

The spectral-amplitude ratios between the 2009 and 2013 UNEs are calculated for six azimuthal ranges, 70–95°, 95–120°, 120–145°, and 145–178° for stations on Japanese islands, and 176–200° and 200–227° for stations on the Korean Peninsula (Fig. 1(a–c)). The spectral-amplitude ratios are found to be similar for all azimuths (Fig. 1(c)). The comparison between the observed and theoretical spectral-amplitude ratios verifies the theoretical UNE source-spectral model. The overshoot parameter in the source-spectral model is *ξ* = 1.05^20^.

### Quasi-observed seismic waveforms

The seismic waveforms for a certain UNE size are calculated by using the waveforms of previous UNEs after correction of the source spectra. Seismic waveforms for an *m*_*b*_5.1 UNE are calculated based on those of the 2009 *m*_*b*_4.7 North Korea UNE. The synthesized waveforms are compared with the observed waveforms of the 2013 *m*_*b*_5.1 North Korean UNE to verify the method (Fig. 1(d)). The synthesized waveforms match the observed waveforms well. The change in frequency content with the UNE size is modeled well. Seismic waveforms for *m*_*b*_7.0 UNE are calculated from both the 2009 *m*_*b*_4.7 and 2013 *m*_*b*_5.1 UNEs. The calculated seismic waveforms based on the 2009 UNE waveforms match those based on the 2013 UNE waveforms well to confirm the validity of the method (Fig. 1(e)). Seismic waveforms for UNEs with magnitudes of 5.0 to 7.6 are synthesized (see, supplementary materials).

### Peak ground motions

The strong ground motions induced by the hypothetical UNEs around Baekdusan are inferred from strong motion attenuation curves that are calibrated to quasi-observed seismic waveforms at known stations. The strong motions attenuate with distance from the sources. The strong motion at a certain distance can be inferred from a reference strong motion attenuation equation. The strong motion attenuation equation is determined by using the seismic records of 55 earthquakes that occurred around the Korean Peninsula from 2001 to 2013. The magnitudes are *M*_*L*_3.0–5.2, and the focal depths are 0.26–19.8 km. Seismic waveforms with signal-to-noise ratios of greater than 2 are selected for analysis. We analyze 7973 horizontal and 3623 vertical record sections from 163 stations. The hypocentral distances are 4–630 km.

**Figure 2:**
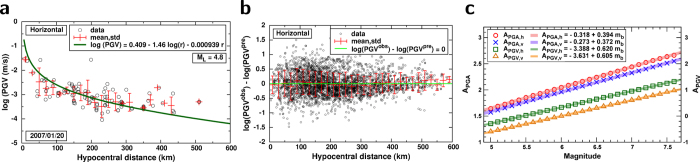
Strong motion attenuation curves. (**a**) An example of the observed horizontal PGVs of an *M*_*L*_ 4.8 earthquake and their fitted attenuation curve. (**b**) Difference between the observed and predicted horizontal PGVs, suggesting good representation of observed PGVs with the PGV attenuation curves. (**c**) Determined event-strength-calibration constants for PGA and PGV attenuation curves for North Korean UNEs as a function of magnitude, presenting strong linear relationships.

**Figure 3:**
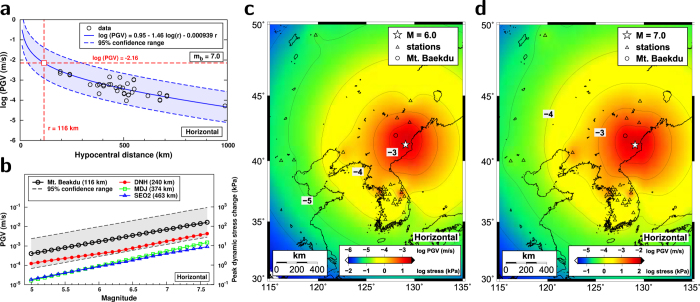
Strong motions induced by UNEs. (**a**) Estimation of PGV on the surface of Baekdusan volcano induced by an *m*_*b*_7.0 UNE. (**b**) Variation of PGVs and equivalent peak dynamic stress changes as a function of magnitude. Spatial distribution of PGVs induced by (**c**) *m*_*b*_ 6.0 and (**d**) *m*_*b*_ 7.0 UNEs. The maps were created by using the software Generic Mapping Tools (http://gmt.soest.hawaii.edu/).

The seismic waveforms are corrected for instrumental responses and bandpass-filtered between 0.01 and 30 Hz. The zero-to-peak ground motions (accelerations, velocities) are measured. A reference strong-motion attenuation curve is determined as a function of distance from the observed peak ground motions. The event-strength-calibration constants adjusting the levels of peak ground motions for hypothetical UNEs are determined from the quasi-observed waveforms (Fig. 2(c)) (see supplementary materials). The strong ground motions on the surface of Baekdusan volcano are determined from the strong motion attenuation curves (Fig. 3(a)).

Hypothetical UNEs with magnitudes (*m*_*b*_) of 5.0–7.6 are expected to produce peak ground velocities (PGVs) on the surface of Baekdusan volcano of 0.00040–0.01610 m/s with a logarithmic 95% confidence range of 0.765 in the horizontal direction, and 0.00025–0.00922 m/s with a logarithmic 95% confidence range of 0.752 in the vertical direction (Fig. 3(b); see, supplementary materials). Also, the peak ground accelerations (PGAs) are 0.02689–0.28030 m/s^2^ with a logarithmic 95% confidence range of 0.868 in the horizontal direction, and 0.01616–0.15308 m/s^2^ with a logarithmic 95% confidence range of 0.860 in the vertical direction. The horizontal and vertical PGVs reach 0.0017 and 0.0010 m/s for an *m*_*b*_ 6.0 UNE, and 0.0069 and 0.0040 m/s for an *m*_*b*_ 7.0 UNE (Fig. 3(c,d)). The horizontal and vertical PGAs reach 0.0683 and 0.0398 m/s^2^ for an *m*_*b*_ 6.0 UNE, and 0.1684 and 0.0917 m/s^2^ for an *m*_*b*_ 7.0 UNE.

### Peak dynamic stress change in magma chamber

**Figure 4:**
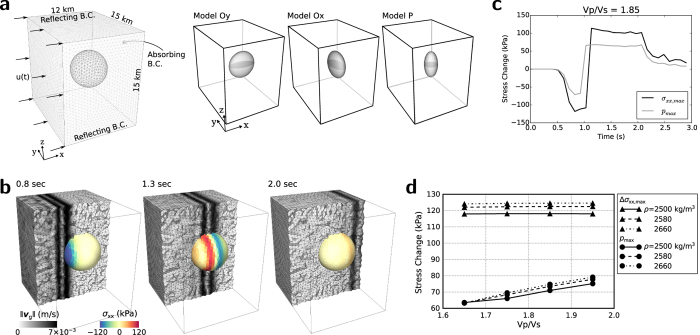
Dynamic stress changes in magma chamber. (**a**) A spherical and three spheroidal magma chamber models in a 3-D domain with annotated boundary conditions and dimensions. A mesh is presented for the magma chamber interface. (**b**) Snapshots of the ground-motion velocities 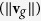 in the crust and horizontal dynamic stress changes (*σ*_*xx*_) in the spherical magma chamber with a *V*_*P*_/*V*_*S*_ ratio of 1.85 at lapse times of 0.8, 1.3 and 2.0 s. (**c**) Peak horizontal dynamic stress changes (*σ*_*xx*,max_) and peak dynamic pressure changes (*p*_*max*_) in the spherical magma chamber with a *V*_*P*_/*V*_*S*_ ratio of 1.85 and density of 2500 kg/m^3^ as a function of time. (**d**) Peak horizontal dynamic stress changes and pressure changes as a function of the *V*_*P*_/*V*_*S*_ ratio in spherical magma chambers with three different densities. The peak horizontal dynamic stress changes are found to be nearly constant, while the peak pressure changes increased with the *V*_*P*_/*V*_*S*_ ratio.

We compute the dynamic stress changes induced in the magma chamber by an *m*_*b*_ 7.0 nuclear explosion with PyLith, which is a finite-element code for dynamic and quasi-static simulations of crustal deformation^42^. We consider an impulsive plane wave incident to the magma chamber in order to estimate the upper bound of the peak dynamic stress change. The plane wave is generated in the left margin of the domain. The strength of the input signal is set to produce transient waves of which peak ground motion is equivalent to that expected from quasi-observed seismic waveforms on the volcano surface (Fig. 4).

A spherical model with a radius of 3 km and three spheroidal models with a flattening of 0.5 are considered for the magma chamber (Fig. 4(a)). We assess the variation in the induced stress depending on the geometry and density of the magma chamber. The densities of the magma chamber are set to be 2500, 2580 and 2660 kg/m^3^ for three different compositions of the magma chamber, representing 50, 30 and 10 vol% of silicic magma compared to the typical continental crust (2700 kg/m^3^)^43^.

To represent various possible states of the magma chamber during an explosive volcanic eruption^44^, we consider four *V*_*P*_/*V*_*S*_ ratios of 1.65, 1.75, 1.85, and 1.95 (Table 1), representing various magma compositions and pore-fluid pressures. The *V*_*P*_/*V*_*S*_ ratio is low in felsic rock and high in mafic rock^45^. Also, the *V*_*P*_/*V*_*S*_ ratio increases with pore-fluid pressure. These *V*_*P*_/*V*_*S*_ ratios are equivalent to Poisson’s ratios of 0.209, 0.258, 0.293, and 0.321.

The transient wavefield comprised compressional pulses satisfying the observed peak ground motions on the volcano surface to induce dynamic stress changes in the magma chamber (Fig. 4(b–d)). The radial peak dynamic stress change in the magma chamber (Δ*σ*_*xx*,*max*_) are found to be constant for *V*_*P*_/*V*_*S*_ ratios, while the peak dynamic pressure change in the magma chamber (*p*_*max*_) increase with the *V*_*P*_/*V*_*S*_ ratio. On the other hand, Δ*σ*_*xx*,*max*_ and *p*_*max*_ generally increase with the density. The spherical magma chamber model produces larger Δ*σ*_*xx*,*max*_ and *p*_*max*_ than the three spheroidal models.

The observations suggest that Δ*σ*_*xx*,*max*_ and *p*_*max*_ are only significant in the model Oy, which are 46 and 83 kPa, respectively, compared to 70 and 124 kPa for the corresponding model with a spherical chamber (*V*_*P*_/*V*_*S*_ = 1.75, density = 2660 kg/m^3^). The other two models, Ox and P produce smaller Δ*σ*_*xx*,*max*_ and *p*_*max*_ than model Oy. This suggests that the spherical model constitutes an environment that produces the upper bound of stress levels by incident seismic waves. The induced peak dynamic pressure change in the spherical magma chamber model ranges between 60 and 80 kPa for various *V*_*P*_/*V*_*S*_ ratios and densities observed in the crust. For the magma chamber with a density of 2500 kg/m^3^, *p*_*max*_ is estimated to be 64 kPa for a *V*_*P*_/*V*_*S*_ ratio of 1.65 and 75 kPa for a *V*_*P*_/*V*_*S*_ ratio of 1.95 (Fig. 4).

## Discussion and Conclusions

The strong motions from hypothetical UNEs were simulated using UNE source-spectral models and seismic records of previous UNEs to avoid the errors associated with uncertainty in crustal models. The method was verified by comparisons between observed and synthetic seismic waveforms for previous UNEs. The peak ground velocities can be converted to peak dynamic stress changes. The peak dynamic stress change was found to linearly increase with the magnitude (Fig. 3(b)). The peak dynamic stress changes on the volcano surface were expected to be 3.8–157.2 kPa in the horizontal direction and 3.4–90.0 kPa in the vertical direction for hypothetical UNEs with magnitudes of 5.0–7.6 (Fig. 3(b–d); see supplementary materials). In particular the horizontal peak dynamic stress changes on the volcano surface reached 16 kPa with an *m*_*b*_ 6.0 UNE and 67 kPa with an *m*_*b*_ 7.0 UNE.

The radial peak dynamic stress change (Δ*σ*_*xx*,*max*_) and induced peak dynamic pressure change in the magma chamber (*p*_*max*_) were found to generally increase with the density. On the other hand, Δ*σ*_*xx*,*max*_ was constant for different *V*_*P*_/*V*_*S*_ ratios, while *p*_*max*_ increased with the *V*_*P*_/*V*_*S*_ ratio. The spherical model constituted the conditions for the upper bounds of Δ*σ*_*xx*,*max*_ and *p*_*max*_ to be reached.

The Δ*σ*_*xx*,*max*_ and *p*_*max*_ values induced by an *m*_*b*_ 7.0 UNE in spherical magma chamber models with densities of 2580 and 2660 kg/m^3^ were about 3 and 5% lower, respectively, than those in the spherical magma chamber with a density of 2500 kg/m^3^. The radial peak dynamic stress change in the magma chamber with a *V*_*P*_/*V*_*S*_ ratio of 1.85 and density of 2500 kg/m^3^ reached the maximum of 118 kPa with averages of around 100–110 kPa during transmission through the chamber (Fig. 4(d)). The overall radial peak dynamic stress changes were generally around 110–130 kPa. The peak dynamic pressure changes were less than 80 kPa for most magma compositions.

Earthquakes are remotely triggered by peak dynamic stress changes of 0.01–1 MPa^46^. The triggering of volcanic eruption is controlled by bubbles that nucleate at different levels of stress (pressure) changes depending on the medium texture^47^. The static pressure in the magma is on the order of several hundred megapascals^47^,^48^,^49^. Reports have suggested that bubbles typically nucleate at magma chamber overpressures of several dozens of MPa^32^,^43^,^47^. Bubble nucleation may start at a pressure change of lower than 1 MPa in a medium with a microlite texture^47^. North Korean nuclear explosions are expected to produce pressure changes of tens to hundreds of kilopascals, causing concern over the possible triggering of volcanic eruption in textured media.

## Methods

### Quasi-observed waveform synthesis

The inter-event distances with respect to the dominant frequencies of regional seismic waves allow us to assume UNEs to be doublet seismic events. The raypath effects to common stations can be assumed to be the same between doublet events. The UNE source spectrum *S*(*f* ) can be represented as a function of the seismic moment^39^,^40^,^41^ (see, supplementary materials). The source-spectral ratio between two events at a common station is given by


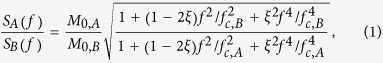


where *S*_*i*_(*f*) is the source spectrum of event *i* (*i* = A, B), *M*_0,*i*_ is the apparent moment of event *i*, *f*_*c*,*i*_ is the corner frequency of event *i*, and *ξ* is the overshoot parameter. The apparent moment and corner frequency can be estimated from linear relationships with the seismic magnitude^41^ (see supplementary materials). The logarithmic apparent moment ratio between two underground nuclear explosions can be written as a function of the magnitude difference:


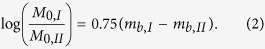


These relationships are particularly effective for UNE sources because they are hardly affected by azimuth-dependent source effects such as directionality and radiation patterns. The spectra of seismic waveforms for an UNE of a certain size can be calculated by using the spectra of observed seismic waveforms for a previous UNE with (1).

### Peak ground motion attenuation curve

The peak ground acceleration (PGA) and peak ground velocity (PGV) attenuate with distance, and generally satisfy the relationship^50^,^51^


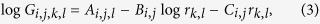


where *G*_*i*,*j*,*k*,*l*_ (*i* = PGA, PGV, *j* = *h*, *v*) is the peak ground motion (PGA or PGV) for the horizontal or vertical component at station *k* for event *l* in a hypocentral distance of *r*_*k*,*l*_, *A*_*i*,*j*,*l*_ is a calibration constant for the event size, *B*_*i*,*j*_ is a calibration constant for the geometrical spreading effect, and *C*_*i*,*j*_ is a calibration constant for anelastic absorption. The PGA is in m/s^2^, the PGV is in m/s^2^, and the distance *r* is in kilometers. The calibration constants are determined so that they yield the minimum squared error (see supplementary materials). The peak ground motion attenuation curve is applied to determine the strength of a strong ground motion at a given distance.

### Estimation of dynamic stress change

The radial peak dynamic stress change *σ*_*r*_ induced by transient seismic waves is given by^52^,^53^


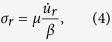


where *μ* is the shear modulus, 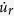 is the peak ground velocity, and *β* is the shear wave velocity. We set the shear modulus at 34.95 GPa and the shear velocity at 3.58 km/s to consider the crustal properties of the Korea Peninsula at a depth of 10 km^45^,^54^ (see supplementary materials).

The dynamic stress changes in the magma chamber is calculated using by PyLith^42^. We consider a spherical model and three spheroidal models for the magma chamber (Fig. 4(a)). The radius of the spherical model is set to 3 km to account for the dimensions of low velocity anomalies revealed in a seismic exploration study^9^. The spheroidal models represent possible geometric orientations of a magma chamber deviating from a symmetric shape (spherical model). The spheroidal models have a flattening of 0.5. The spheroidal models comprise one prolate spheroid (P) and two oblate spheroids for which the symmetry axes are parallel with the *x* (model Ox) or *y* (Oy) axis. We consider a 12 × 15 × 15 km rectangular domain to represent a homogeneous medium that sufficiently accommodates a magma chamber model in the center.

We design a situation in which an impulsive plane wave approaches the magma chamber in order to estimate the peak dynamic stress change. The plane wave is generated in the left-hand side of the domain. The right-hand side boundary of the domain is set to an absorbing boundary condition. The boundary-normal displacement component is set to be zero on the upper and lower boundaries. The initial stress field is set to be zero over the domain, and the gravitational body force is not applied. The magma chamber is treated as an elastic body to estimate the possible upper limit of peak stress changes. The seismic velocities of the medium surrounding the magma chamber are *V*_*P*_ = 6.17 km/s and *V*_*S*_ = 3.57 km/s, and the density was 2700 kg/m^3^.

## Additional Information

**How to cite this article**: Hong, T.-K. *et al.* Prediction of ground motion and dynamic stress change in Baekdusan (Changbaishan) volcano caused by a North Korean nuclear explosion. *Sci. Rep.* **6**, 21477; doi: 10.1038/srep21477 (2016).

## Supplementary Material

Supplementary Information

## Figures and Tables

**Table 1:** Medium properties of four magma chamber models: *V*_*P*_/*V*_*S*_ ratio, Poisson’s ratio (*ν*), seismic-wave velocities (*V*_*P*_, *V*_*S*_), and bulk and shear moduli (*K*, *μ*).

Model	*V*_*P*_/*V*_*S*_	*V*_*P*_(km/s)	*V*_*S*_(km/s)	*K*(GPa)	*μ*(GPa)	*ν*
M1	1.65	5.90	3.58	44.3	32.0	0.209
M2	1.75	5.90	3.37	49.2	28.4	0.258
M3	1.85	5.90	3.19	53.1	25.4	0.293
M4	1.95	5.90	3.03	56.4	23.0	0.321
