# Supplementary material for: Prediction of ground motion and dynamic stress change in Baekdusan (Changbaishan) volcano caused by a North Korean nuclear explosion
Source: Sci Rep. 2016 Feb 17;6:21477. doi: 10.1038/srep21477 (PMC4756380; doi:10.1038/srep21477)
Supplement: Supplementary Information [file srep21477-s1.pdf]

Supplementary Materials

Prediction of ground motion and dynamic stress change in Baekdusan (Changbaishan) volcano caused by a North Korean nuclear explosion

Tae-Kyung Hong[1], Eunseo Choi[2], Seongjun Park[1], and Jin Soo Shin[3]

Affiliations and addresses:

[1]Yonsei University, Department of Earth System Sciences, 50 Yonsei-ro, Seodaemun-gu Seoul 120-749, South Korea (tkhong@yonsei.ac.kr, seongjunpark@yonsei.ac.kr)

[2] Center for Earthquake Research and Information, University of Memphis, Memphis, Tennessee 38152, USA (echoi2@memphis.edu)

[3] Earthquake Research Center, Korea Institute of Geoscience and Mineral Resources, 92 Gwahang-no, Yuseong-gu, Daejeon 305-350, South Korea (jinsoo@kigam.re.kr)

Table 1. Seismic source information of the North Korean nuclear explosions

ID	date	time (UTC)	lat (°N)	lon (°E)	$m_b(Lg)$	m_b	M_w	M_0 ($\times 10^{15}$ Nm)
NK2006	2006/10/09	01:35:28	41.287	129.108	3.93	4.3		0.29
NK2009	2009/05/25	00:54:43.2	41.294	129.077	4.53	4.7	4.5	6.14
NK2013	2013/02/12	02:57:51.2	41.292	129.073	4.91	5.1	4.7	13.7

Korean Peninsula

The Korean Peninsula is located in the far eastern continental margin of the Eurasian plate that experienced complex continental collision and rifting. The peninsula was formed by a series of continental collision and rifting from late Permian to mid-Miocene (Jolivet et al., 1994; Chough et al., 2000; Oh, 2006). The rifting opened the East Sea (Sea of Japan), constructing a continent-ocean transitional crustal structure off the east coast of the Korean Peninsula (Jolivet et al, 1994; Hong et al., 2008). The successive complex tectonic evolutions constructed complicated crustal structures. The inland peninsula and the Yellow Sea regions display continental crusts with thicknesses of 28-38 km, while the East Sea region has a transitional oceanic crust with thicknesses less than 20 km (Hong et al., 2008; He and Hong, 2010).

North Korean underground nuclear explosions

North Korea conducted three underground nuclear explosion (UNE) tests in 2006, 2009 and 2013 (Table **??**; Hong and Rhie, 2009; Shin et al., 2010; Zhao et al., 2012; Zhang and Wen, 2013; Zhao et al., 2014). The UNEs were well recorded in regional distances (Fig. **??**).

Synthesis of quasi-observed waveforms

The UNE source spectrum $S(f)$ can be represented by (Mueller and Murphy, 1971; Sereno *et al.*, 1988; Xie and Patton, 1999)

$$S(f) = \frac{M_0}{4\pi\rho_s v_s^3 \sqrt{1 + (1-2\xi) f^2/f_c^2 + \xi^2 f^4/f_c^4}} \qquad (1)$$

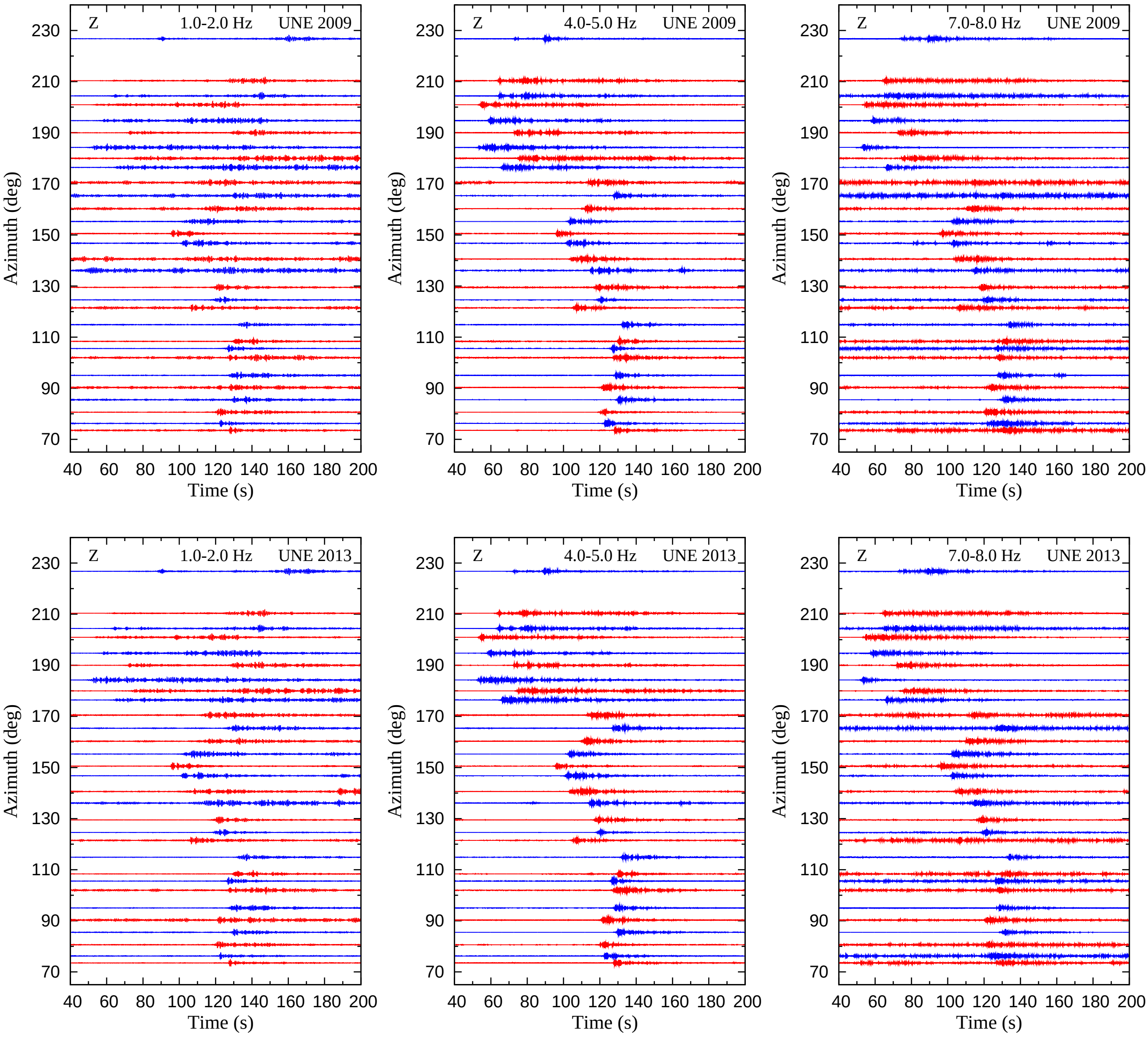

Figure 1. Seismic waveforms of the 2009 and 2013 North Korea UNEs at frequency bands of 1.0-2.0 Hz, 4.-5.0 Hz, and 7.0-8.0 Hz. The first-arrival P waves are clearly identified.

where M_0 is the apparent moment in Nm, ξ is the overshoot parameter, f_c is the corner frequency, ρ_s is the density in the source region, and v_s is the velocity of the phase in the source region. The source-spectral ratio between two events is given by

$$\frac{S_A(f)}{S_B(f)} = \frac{M_{0,A}}{M_{0,B}} \sqrt{\frac{1 + (1 - 2\xi) f^2 / f_{c,B}^2 + \xi^2 f^4 / f_{c,B}^4}{1 + (1 - 2\xi) f^2 / f_{c,A}^2 + \xi^2 f^4 / f_{c,A}^4}}. \tag{2}$$

where $S_i(f)$ is the source spectrum of event i (i =A,B), $M_{0,i}$ is the apparent moment of event i, and $f_{c,i}$ is the corner frequency of event i. The low-frequency asymptote of the source-spectral

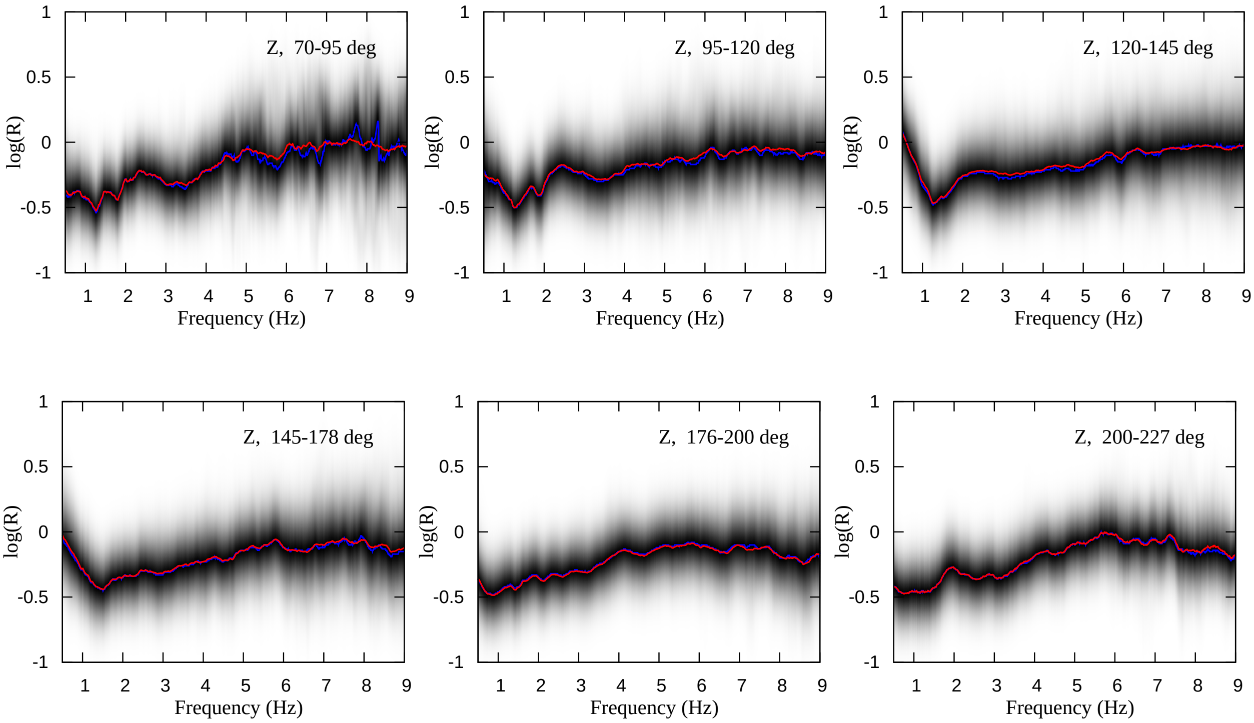

Figure 2. Spectral-amplitude ratios between the 2009 and 2013 UNEs at stations in azimuthal ranges of 70-95°, 95-120°, 120-145°, 145-178°, 176-200°, and 200-227°. The mean variations are marked with red lines.

ratio is equivalent to the apparent moment ratio. There is inherent trade-offs between apparent moment and attenuation factors exist (Hong, 2013) (Fig. **??**).

The logarithmic apparent moment for regional phase have linear relationships with magnitude and logarithmic corner frequency (Xie and Patton, 1999). The logarithmic apparent moment for Pn satisfies

$$\log M_{0,i} = 9.53 + 1.16\, m_{b,i},$$
$$\log M_{0,i} = 18.34 - 4.73 \log f_{c,i}, \qquad (3)$$

where $m_{b,i}$ is the body-wave magnitude of event i. From equation (**??**), the relationship between the magnitude and corner frequency is given by

$$\log f_{c,i} = -0.25\, m_{b,i} + 1.86. \qquad (4)$$

The logarithmic apparent moment ratio between two underground nuclear explosions can be

expressed as a function of magnitude differences:

$$\log\left(\frac{M_{0,I}}{M_{0,II}}\right) = 0.75\,(m_{b,I} - m_{b,II})\,. \tag{5}$$

Quasi-observed seismic waveforms for a hypothetical North Korean UNE can be calculated based on the seismic waveforms of a previous North Korean UNE using equation (**??**). Quasi-observed waveforms for m_b 5.1 UNE are calculated based on the seismic waveforms of the 2009 m_b 4.7 UNE, and are compared with the seismic waveforms of the 2013 m_b 5.1 UNE (Fig. **??**). Also, quasi-observed waveforms for m_b 6.0 and 7.0 are calculated based on both the 2009 and 2013 UNE records. The waveforms match well each other, verifying the methodology.

The approach is applied to UNEs from other test site. Seismic waveforms for Balapan UNEs at station WMQ are analyzed (Hong, 2010). Quasi-observed seismic waveforms are calculated based on a Balapan UNE, and are compared with the observed seismic waveforms for a nearby UNE (Fig. **??**). The waveforms match well each other.

Peak ground motion attenuation

The peak ground acceleration (PGA) and peak ground velocity (PGV) attenuate with distance, and generally satisfy a relationship (e.g., Atkinson and Boore, 1995; Marin et al., 2004)

$$\log G_{i,j,k,l} = A_{i,j,l} - B_{i,j}\,\log\,r_{k,l} - C_{i,j}\,r_{k,l}, \tag{6}$$

where $G_{i,j,k,l}$ (i =PGA, PGV, $j = h, v$) is peak ground motion (PGA or PGV) in horizontal or vertical component at station k for event l in the hypocentral distance of $r_{k,l}$, $A_{i,j,l}$ is a calibration constant for event size, $B_{i,j}$ is a calibration constant for geometrical spreading effect, and $C_{i,j}$ is a calibration constant for anelastic absorption. The PGA is in m/s^2, the PGV is in m/s^2, and the distance r is in km. The squared error between observed and theoretical peak ground motion (PGA, PGV) is given by

$$\phi_{i,j} = \sum_{l=1}^{N_e}\sum_{k=1}^{N_{s,l}}\left[\log G^{obs}_{i,j,k,l} - (A_{i,j,l} - B_{i,j}\log r_{k,l} - C_{i,j}\,r_{k,l})\right]^2, \tag{7}$$

where $\phi_{i,j}$ (i=PGA, PGV, $j = h, v$) is the squared error of peak ground motion (PGA or PGV) in horizontal or vertical component, $G^{obs}_{i,j,k,l}$ is the observed peak ground motion, N_e is

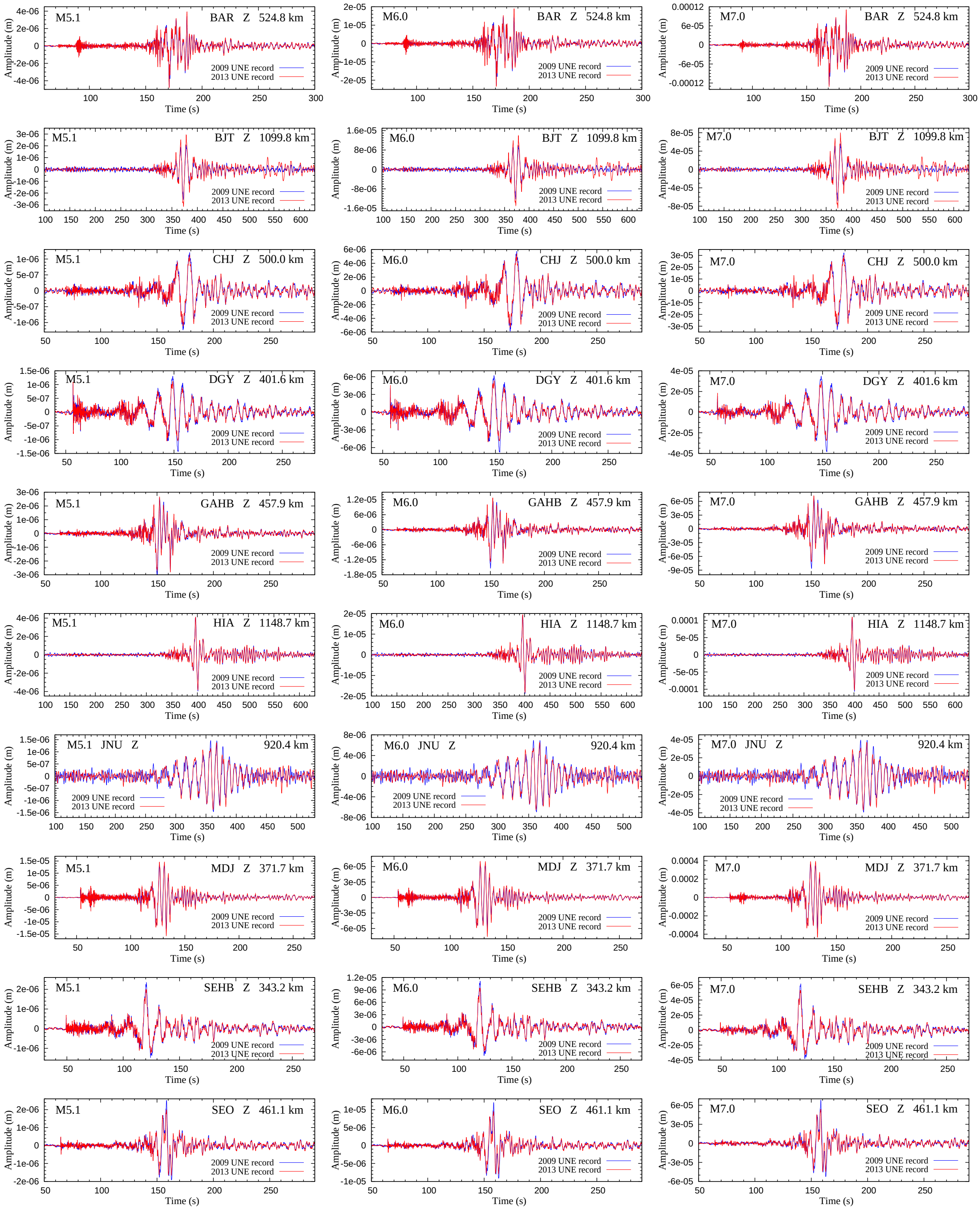

Figure 3. Synthesis of quasi-observed seismic waveforms for UNEs of m_b 5.1, 6.0, and 7.0. The quasi-observed seismic waveforms for m_b 5.1 are compared with the observed seismic waveforms for the 2013 UNE. Quasi-observed waveforms are synthesized based on both the 2009 and 2013 UNEs, presenting good agreement each other.

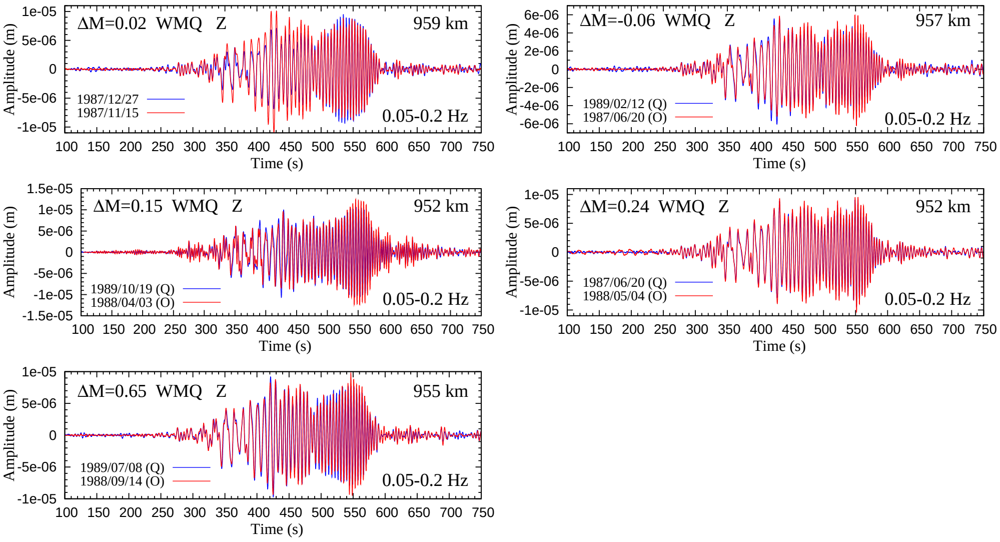

Figure 4. Comparison of quasi-observed seismic waveforms with the observed seismic waveforms at station WMQ for underground nuclear explosions in the Balapan nuclear test site. The magnitude differences vary between 0.02 and 0.65 in magnitude unit. The epicentral distances are 952-959 km.

the number of events, $N_{s,l}$ is the number of stations for event l. The calibration constants for geometrical spreading and aleastic absorption are determined so that they yield the minimum squared error:

$$\frac{\partial \phi_{i,j}}{\partial B_{i,j}} = \frac{\partial \phi_{i,j}}{\partial C_{i,j}} = 0. \tag{8}$$

Once the calibration constants $B_{i,j}$ and $C_{i,j}$ are determined, the calibration constant for event size is determined by

$$A_{i,j,l} = \frac{1}{N_{s,l}} \sum_{k=1}^{N_{s,l}} \left(\log G_{i,j,k,l}^{obs} + B_{i,j} \log r_{k,l} + C_{i,j}\, r_{k,l} \right). \tag{9}$$

The constants for PGA attenuation curves are found to be $B_{PGA,h} = -1.44$, $B_{PGA,v} = -1.54$, $C_{PGA,h} = -0.00211$, and $C_{PGA,v} = -0.00164$, and those for PGV attenuation curves are $B_{PGV,h} = -1.46$, $B_{PGV,v} = -1.52$, $C_{PGV,h} = -0.000939$, and $C_{PGV,v} = -0.000739$. The peak ground motions agree well with the regression curves. The residuals between the observed PGAs and regression curves are clustered around zero. It is observed that the source-strength

constants satisfy

$$A_{PGA,h} = -0.318 + 0.394m_b, \quad A_{PGA,v} = -0.273 + 0.372m_b,$$
$$A_{PGV,h} = -3.388 + 0.620m_b, \quad A_{PGV,v} = -3.631 + 0.605m_b. \tag{10}$$

The observed PGVs are compared with the PGV attenuation curves for three earthquakes (Fig. ??). The observed PGVs agree well with the attenuation curves. Also, residuals between observed PGVs and theoretical PGVs for all earthquakes are examined (Fig. ??). The theoretical PGVs are calculated from the PGV attenuation curve. The residuals are clustered around zero, verifying the PGV attenuation curves. Similarly, we analyze for PGAs, and find good agreement between the observed and theoretical PGAs (Figs. ??, ??).

Dynamic stress change

The peak radial dynamic strain, ϵ_r, induced by transient seismic waves can be calculated by (Gomberg and Davis, 1996):

$$\epsilon_r = \frac{\dot{u_r}}{\beta}, \tag{11}$$

where $\dot{u_r}$ is the peak ground velocity, and β is the shear wave velocity. The induced peak dynamic stress change, σ_r, is given by (Hill et al., 1993)

$$\sigma_r = \mu\frac{\dot{u_r}}{\beta}, \tag{12}$$

where μ is the shear modulus. We set the shear modulus of 34.95 GPa and the shear velocity of 3.58 km/s considering the crustal properties at the depth of 10 km in the Korea Peninsula (Chang and Baag, 2006; Jo and Hong, 2013).

The expected PGVs or PGAs on the ground of Baekdu volcano by hypothetical UNEs with magnitudes of 5.0-7.6 are calculated from the PGV or PGA attenuation curves (Figs. ??, ??, ??, ??). The strong motions (PGVs, PGAs) appear to increase with magnitude (Figs. ??, ??). The peak ground velocities (PGVs) on the ground of volcano reach 0.00040-0.01610 m/s with logarithmic 95 % confidence range of 0.765 in horizontal direction, and 0.00025-00922 m/s with logarithmic 95 % confidence range of 0.752 in vertical direction for the hypothetical

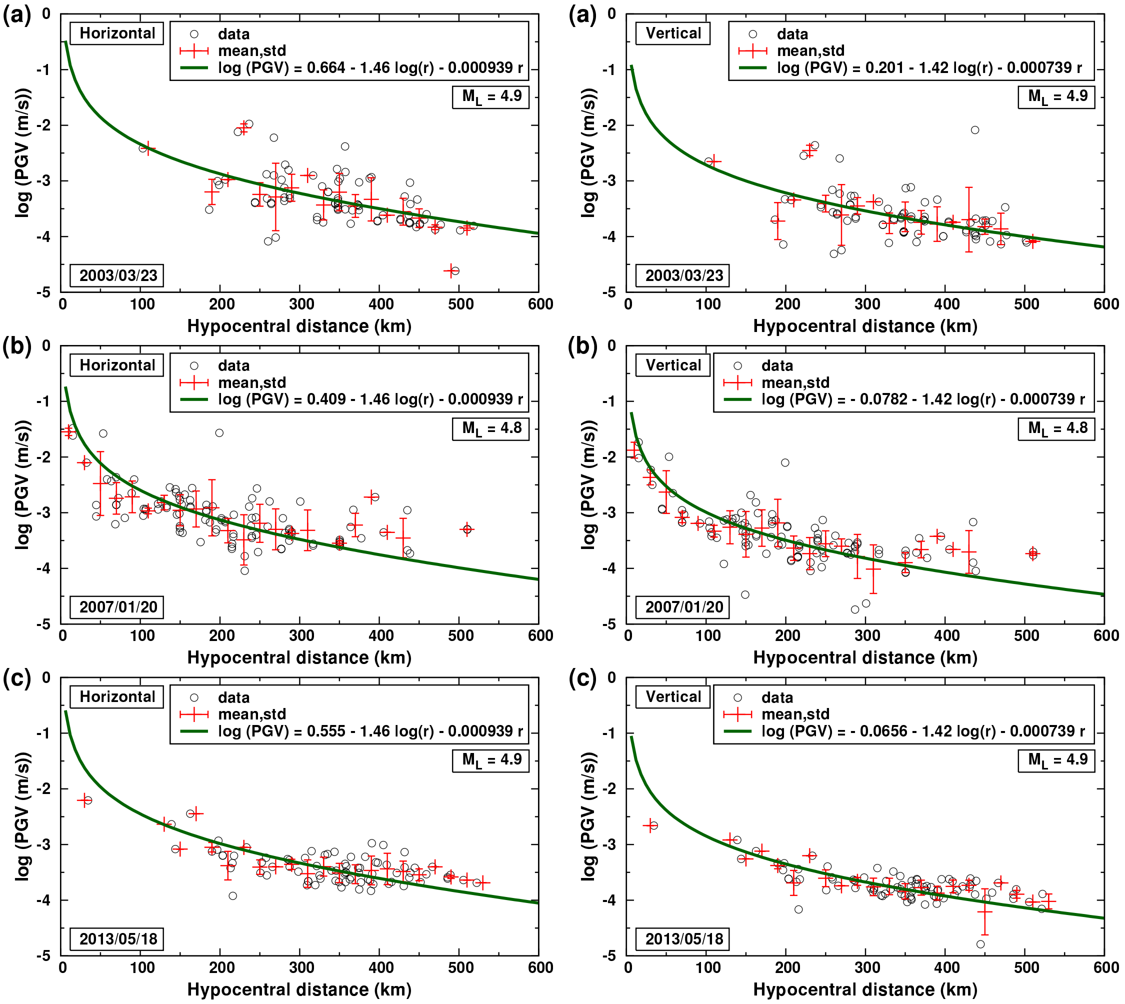

Figure 5. Variation of horizontal and vertical PGVs with distance, and comparison with PGV attenuation curves. Seismic waveforms from three earthquakes with magnitudes of M_L 4,8 and 4.9 are analyzed. The PGV attenuation curves agree well with the observed PGVs.

UNEs (Tables **??**, **??**). Also, the peak ground accelerations (PGAs) are 0.02689-0.28030 m/s^2 with logarithmic 95 % confidence range of 0.868 in horizontal direction, and 0.01616-0.15308 m/s^2 with logarithmic 95 % confidence range of 0.860 in vertical direction (Tables **??**, **??**). The horizontal and vertical PGVs reach 0.0017 and 0.0010 m/s for an m_b 6.0 UNE, and 0.0069 and 0.0040 m/s for an m_b 7.0 UNE (Fig. **??**). Also, the horizontal and vertical PGAs reach 0.0683 and 0.0398 m/s^2 for an m_b 6.0 UNE, and 0.1684 and 0.0917 m/s^2 for an m_b 7.0 UNE (Fig. **??**).

Table 2. Expected strengths ($\bar{B}$) and 95 % confidence ranges (B_1, B_2) of horizontal peak ground velocities, peak ground accelerations, and dynamic stress changes on the ground of Baekdu volcano by detonation of UNEs with magnitudes of m_b 5.0-7.6.

mag	peak ground velocity (m/s)			peak ground acceleration (m/s^2)			dynamic stress change (kPa)		
(m_b)	$\bar{B}$	B_1	B_2	$\bar{B}$	B_1	B_2	$\bar{B}$	B_1	B_2
5.0	0.00040	0.00007	0.00231	0.02689	0.00364	0.19856	3.866	0.664	22.518
5.1	0.00046	0.00008	0.00266	0.02973	0.00403	0.21952	4.460	0.766	25.979
5.2	0.00053	0.00009	0.00307	0.03255	0.00441	0.24035	5.141	0.883	29.944
5.3	0.00061	0.00010	0.00353	0.03583	0.00485	0.26455	5.918	1.016	34.468
5.4	0.00070	0.00012	0.00406	0.03925	0.00532	0.28982	6.808	1.169	39.650
5.5	0.00080	0.00014	0.00468	0.04368	0.00592	0.32250	7.835	1.345	45.633
5.6	0.00094	0.00016	0.00547	0.04781	0.00648	0.35296	9.162	1.573	53.363
5.7	0.00109	0.00019	0.00634	0.05229	0.00708	0.38606	10.630	1.825	61.912
5.8	0.00125	0.00021	0.00729	0.05715	0.00774	0.42192	12.219	2.098	71.169
5.9	0.00144	0.00025	0.00839	0.06245	0.00846	0.46111	14.052	2.413	81.843
6.0	0.00166	0.00028	0.00964	0.06832	0.00925	0.50438	16.162	2.775	94.134
6.1	0.00190	0.00033	0.01108	0.07481	0.01013	0.55233	18.565	3.187	108.128
6.2	0.00219	0.00038	0.01274	0.08116	0.01099	0.59922	21.341	3.664	124.295
6.3	0.00252	0.00043	0.01468	0.08899	0.01205	0.65705	24.592	4.222	143.232
6.4	0.00294	0.00050	0.01712	0.09747	0.01320	0.71961	28.693	4.926	167.114
6.5	0.00340	0.00058	0.01982	0.10663	0.01444	0.78726	33.207	5.701	193.409
6.6	0.00393	0.00068	0.02291	0.11597	0.01571	0.85624	38.389	6.591	223.590
6.7	0.00454	0.00078	0.02643	0.12723	0.01723	0.93933	44.293	7.605	257.977
6.8	0.00522	0.00090	0.03043	0.13980	0.01893	1.03214	50.995	8.756	297.012
6.9	0.00601	0.00103	0.03498	0.15352	0.02079	1.13343	58.617	10.064	341.401
7.0	0.00688	0.00118	0.04010	0.16841	0.02281	1.24338	67.196	11.537	391.369
7.1	0.00786	0.00135	0.04578	0.18655	0.02527	1.37731	76.722	13.173	446.849
7.2	0.00926	0.00159	0.05391	0.20420	0.02766	1.50762	90.334	15.510	526.136
7.3	0.01078	0.00185	0.06276	0.22303	0.03021	1.64664	105.176	18.058	612.578
7.4	0.01221	0.00210	0.07111	0.24296	0.03291	1.79383	119.162	20.459	694.035
7.5	0.01386	0.00238	0.08071	0.26416	0.03578	1.95032	135.248	23.221	787.730
7.6	0.01611	0.00277	0.09381	0.28030	0.03796	2.06949	157.208	26.992	915.630

Table 3. Expected strengths ($\bar{B}$) and 95 % confidence ranges (B_1, B_2) of vertical peak ground velocities, peak ground accelerations, and dynamic stress changes on the ground of Baekdu volcano by detonation of UNEs with magnitudes of m_b 5.0-7.6.

mag	peak ground velocity (m/s)			peak ground acceleration (m/s^2)			dynamic stress change (kPa)		
(m_b)	$\bar{B}$	B_1	B_2	$\bar{B}$	B_1	B_2	$\bar{B}$	B_1	B_2
5.0	0.00025	0.00004	0.00139	0.01616	0.00223	0.11702	2.412	0.427	13.615
5.1	0.00028	0.00005	0.00160	0.01779	0.00246	0.12882	2.762	0.489	15.593
5.2	0.00032	0.00006	0.00183	0.01952	0.00270	0.14132	3.161	0.560	17.841
5.3	0.00037	0.00007	0.00209	0.02144	0.00296	0.15524	3.616	0.641	20.408
5.4	0.00042	0.00008	0.00240	0.02352	0.00325	0.17027	4.144	0.734	23.389
5.5	0.00049	0.00009	0.00275	0.02576	0.00356	0.18653	4.757	0.843	26.850
5.6	0.00056	0.00010	0.00316	0.02820	0.00389	0.20413	5.469	0.969	30.867
5.7	0.00064	0.00011	0.00364	0.03082	0.00426	0.22316	6.295	1.115	35.531
5.8	0.00074	0.00013	0.00418	0.03362	0.00464	0.24339	7.234	1.282	40.834
5.9	0.00085	0.00015	0.00480	0.03658	0.00505	0.26482	8.298	1.470	46.836
6.0	0.00097	0.00017	0.00550	0.03982	0.00550	0.28831	9.513	1.685	53.695
6.1	0.00112	0.00020	0.00632	0.04326	0.00597	0.31317	10.920	1.935	61.640
6.2	0.00128	0.00023	0.00724	0.04704	0.00650	0.34054	12.519	2.218	70.663
6.3	0.00147	0.00026	0.00831	0.05115	0.00706	0.37031	14.370	2.546	81.110
6.4	0.00170	0.00030	0.00961	0.05620	0.00776	0.40689	16.625	2.945	93.841
6.5	0.00197	0.00035	0.01112	0.06110	0.00844	0.44234	19.228	3.407	108.535
6.6	0.00227	0.00040	0.01283	0.06652	0.00919	0.48159	22.192	3.932	125.263
6.7	0.00262	0.00046	0.01479	0.07238	0.01000	0.52405	25.576	4.531	144.363
6.8	0.00302	0.00053	0.01703	0.07858	0.01085	0.56894	29.450	5.218	166.232
6.9	0.00347	0.00061	0.01957	0.08523	0.01177	0.61708	33.846	5.996	191.042
7.0	0.00398	0.00071	0.02249	0.09174	0.01267	0.66419	38.880	6.888	219.458
7.1	0.00456	0.00081	0.02576	0.09980	0.01378	0.72251	44.534	7.890	251.373
7.2	0.00522	0.00093	0.02947	0.10855	0.01499	0.78587	50.965	9.029	287.674
7.3	0.00596	0.00106	0.03365	0.11841	0.01635	0.85725	58.189	10.309	328.448
7.4	0.00679	0.00120	0.03834	0.12906	0.01783	0.93438	66.303	11.746	374.243
7.5	0.00808	0.00143	0.04560	0.14068	0.01943	1.01851	78.852	13.970	445.081
7.6	0.00922	0.00163	0.05205	0.15308	0.02114	1.10826	90.006	15.946	508.037

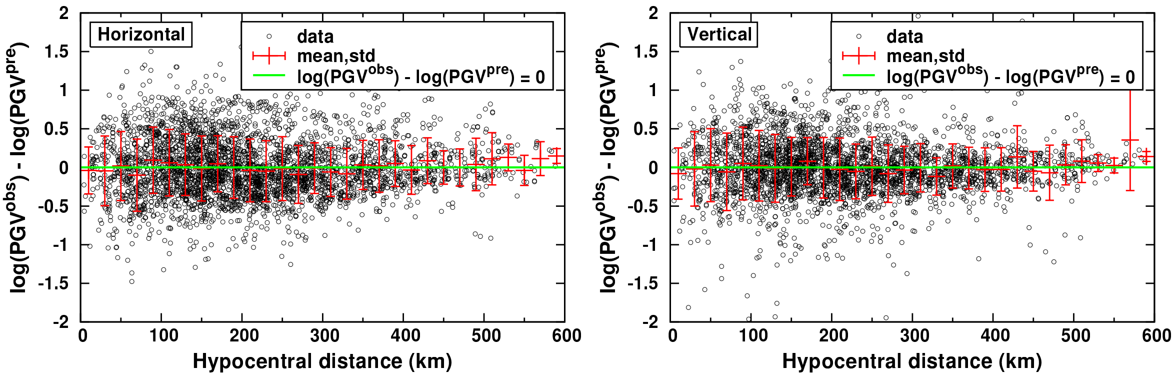

Figure 6. Residuals between the observed and predicted PGVs in horizonal and vertical components as a function of distance. The residuals are clustered around zero.

The dynamic stress changes induced in the magma chamber by nuclear explosion is calculated using PyLith (Aagaard et al., 2013). We assume a spherical magma chamber with a radius of 3 km radius in a 12 × 15 × 15 km rectangular domain representing the crust (Fig. 4(a)). We consider an impulsive plane wave approaching to the magma chamber, aiming to estimate the peak dynamic stress change. The plane wave is generated in the left-hand side of domain, which is given by

$$u(t) = u_0 \frac{t}{\tau} \exp\left(-\frac{t}{\tau}\right), \tag{13}$$

where u is the boundary-normal component of displacement, t is time and u_0 and τ are set to be 0.4 m and 1.5 sec. The right-hand side boundary of the domain is treated by an absorbing boundary condition. The boundary-normal displacement component is set to be zero on the upper and lower boundaries. The initial stress field is set to be zero over the domain, and the gravitational body force is not applied.

References

Aagaard, B.T., M. G. Knepley, and C. A. Williams (2013). A domain decomposition approach to implementing fault slip in finite-element models of quasi-static and dynamic crustal deformation, Journal of Geophysical Research, 118 (6), 3059-3079.

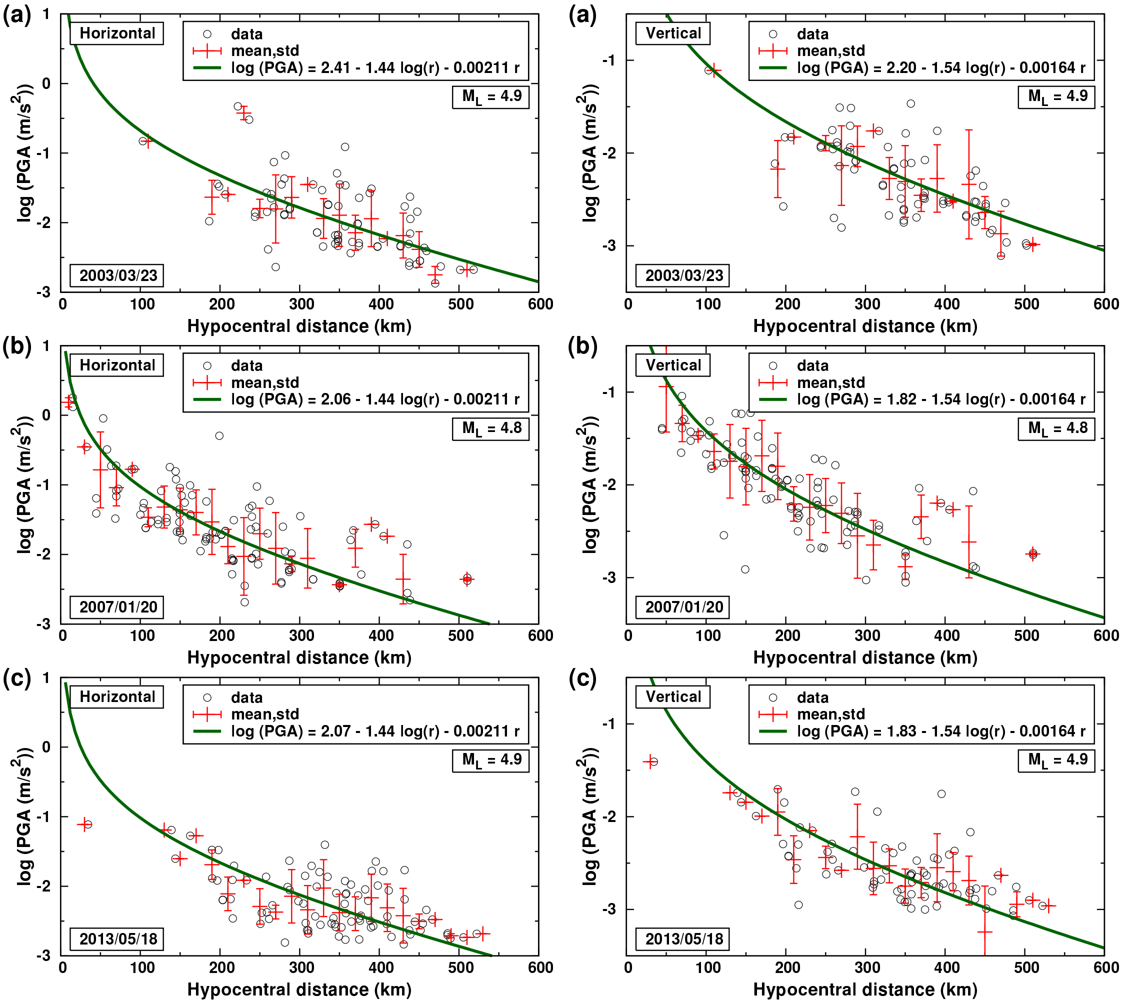

Figure 7. Variation of horizontal and vertical PGAs with distance, and comparison with PGA attenuation curves. Seismic waveforms from three earthquakes with magnitudes of M_L 4,8 and 4.9 are analyzed. The PGA attenuation curves agree well with the observed PGAs.

Atkinson, M., and D. M. Boore (1995). Ground-motion relations for eastern North America, Bulletin of the Seismological Society of America 85, 17-30.

Chang, S.-J., and C.-E. Baag (2006). Crustal structure in southern Korea from joint analysis of regional broadband waveforms and travel times. Bulletin of the Seismological Society of America 96, 856-870.

Chough, S.K., S.-T. Kwon, J.-H. Ree, and D.-K. Choi (2000). Tectonic and sedimentary evolution of the Korean Peninsula: a review and new view, Earth-Science Reviews, 52, 175-235.

Gomberg, J. and S. Davis (1996). Stress/strain changes and triggered seismicity at The Geysers, California, Journal of Geophysical Research, 101 (B1), 733-749.

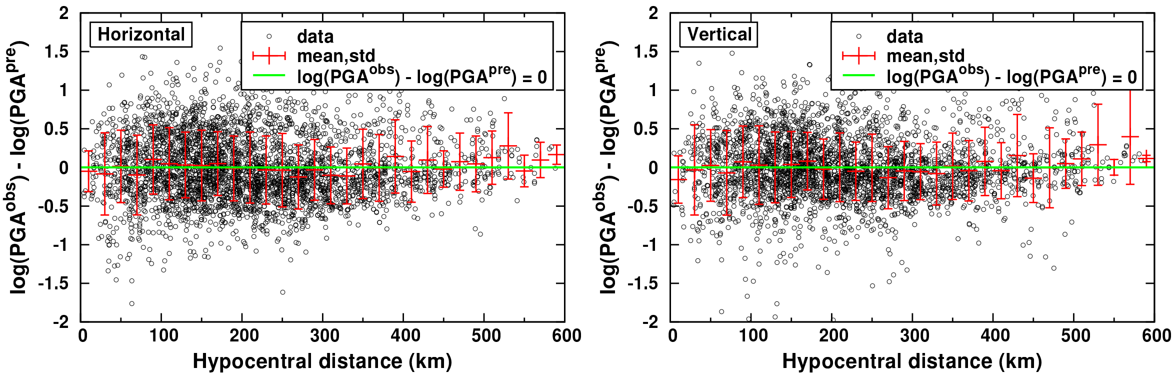

Figure 8. Residuals between the observed and predicted PGAs in horizonal and vertical components as a function of distance. The residuals are clustered around zero.

He, X. and T.-K. Hong (2010). Evidence for strong ground motion by waves refracted from the Conrad discontinuity, Bulletin of the Seismological Society of America, 100 (3), 1370-1374.

Hill, D.P., P. A. Reasenberg, A. Michael, W. J. Arabaz, G. Beroza, D. Brumbaugh, J. N. Brune, R. Castro, S. Davis, D. dePolo, W. L. Ellsworth, J. Gomberg, S. Harmsen, L. House, S. M. Jackson, M. J. S. Johnston, L. Jones, R. Keller, S. Malone, L. Munguia, S. Nava, J. C. Pechmann, A. Sanford, R. W. Simpson, R. B. Smith, M. Stark, M. Stickney, A. Vidal, S. Walter, V. Wong, J. Zollweg (1993). Seismicity remotely triggered by the magnitude 7.3 Landers, California, earthquake, Science, 260, 1617-1623.

Hong, T.-K. (2010). Regional and teleseismic analysis of underground nuclear explosion waveforms and constraints for shear-wave excitation mechanisms, Journal of Geophysical Research, 115, B06306, doi:10.1029/2009JB006368.

Hong, T.-K. (2013). Seismic discrimination of the 2009 North Korean nuclear explosion based on regional source spectra, Journal of Seismology, 17, 753-769

Hong, T.-K., C.-E. Baag, H. Choi, and D.-H. Sheen (2008). Regional seismic observations of the 9 October 2006 underground nuclear explosion in North Korea and the influence of crustal structure on regional phases, Journal of Geophysical Research, 113, B03305, doi:10.1029/2007JB004950.

Hong, T.-K. and J. Rhie (2009). Regional source scaling of the 9 October 2006 underground nuclear explosion in North Korea, Bulletin of the Seismological Society of America, 99 (4), 2523-2540.

Jo, E., and T.-K. Hong (2013). Vp/Vs ratios in the upper crust of the southern Korean Peninsula and their correlations with seismic and geophysical properties, Journal of Asian Earth Sciences, 66, 204-214.

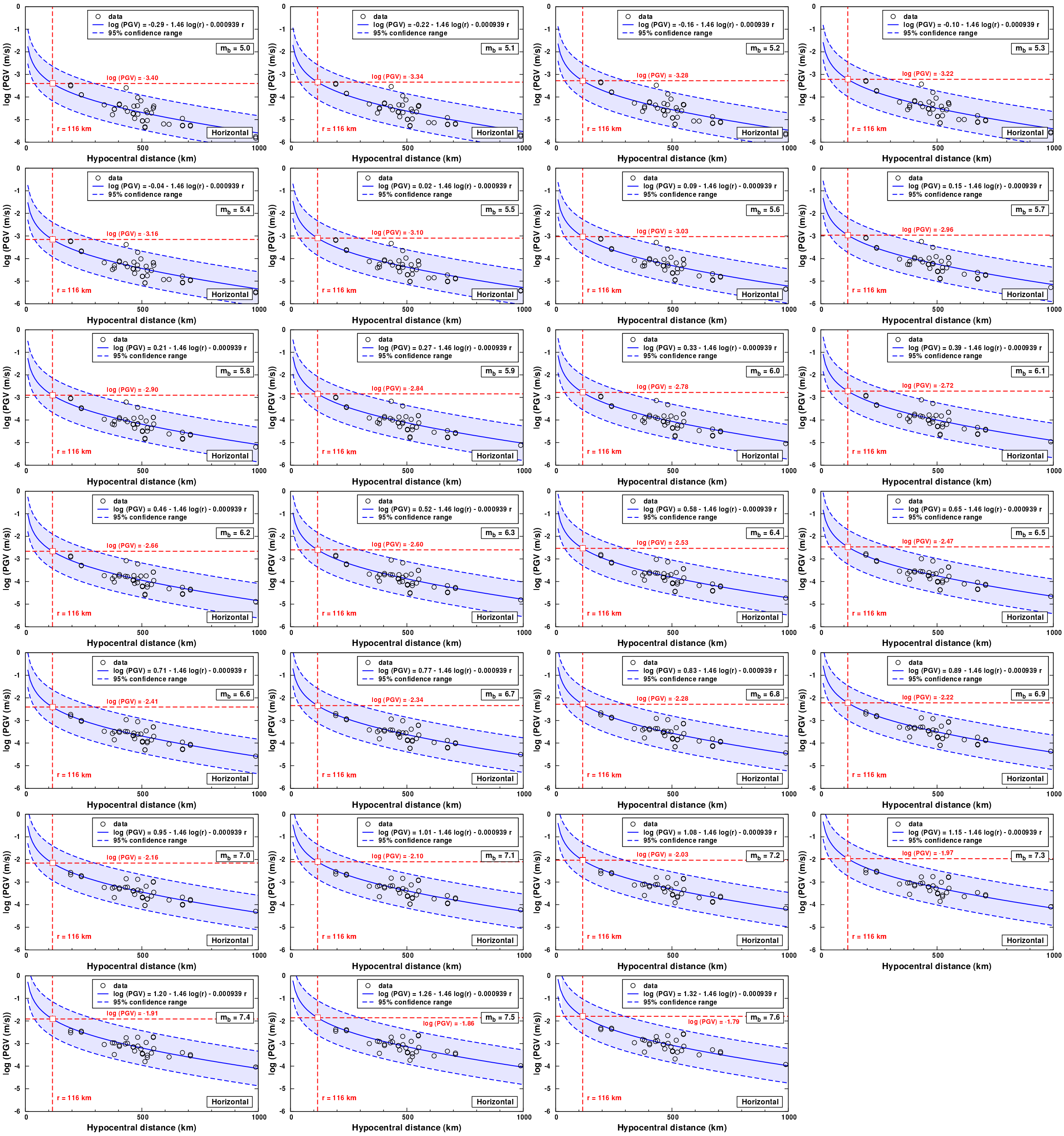

Figure 9. Determination of horizontal PGVs on the ground of Baekdu volcano for UNEs with magnitudes of m_b 5.0-7.6. The horizontal PGV attenuation curves are presented in solid blue lines with 95 % confidence ranges in shade of blue.

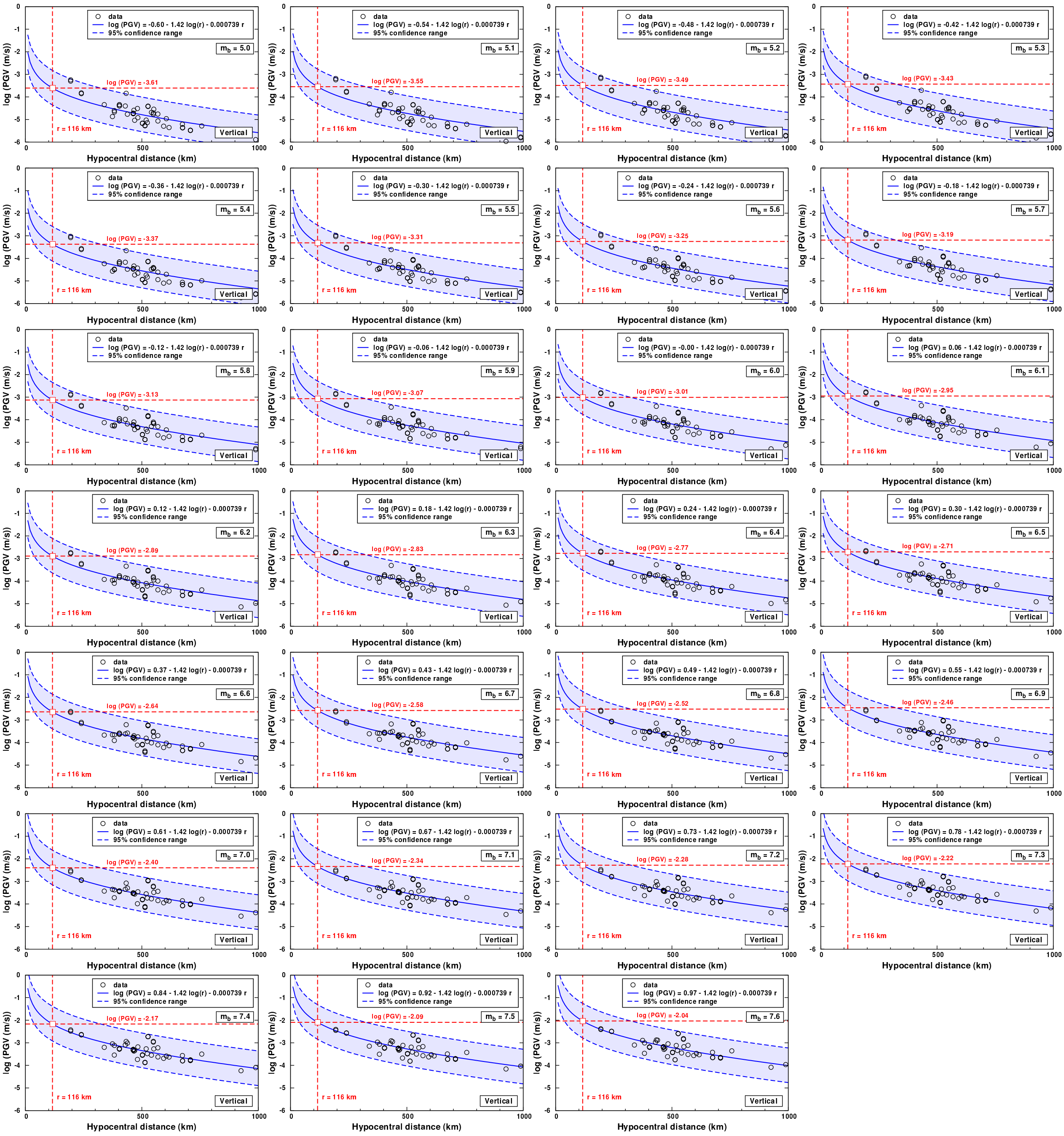

Figure 10. Determination of vertical PGVs on the ground of Baekdu volcano for UNEs of m_b 5.0-7.6. The vertical PGV attenuation curves are presented in solid blue lines with 95 % confidence ranges in shade of blue.

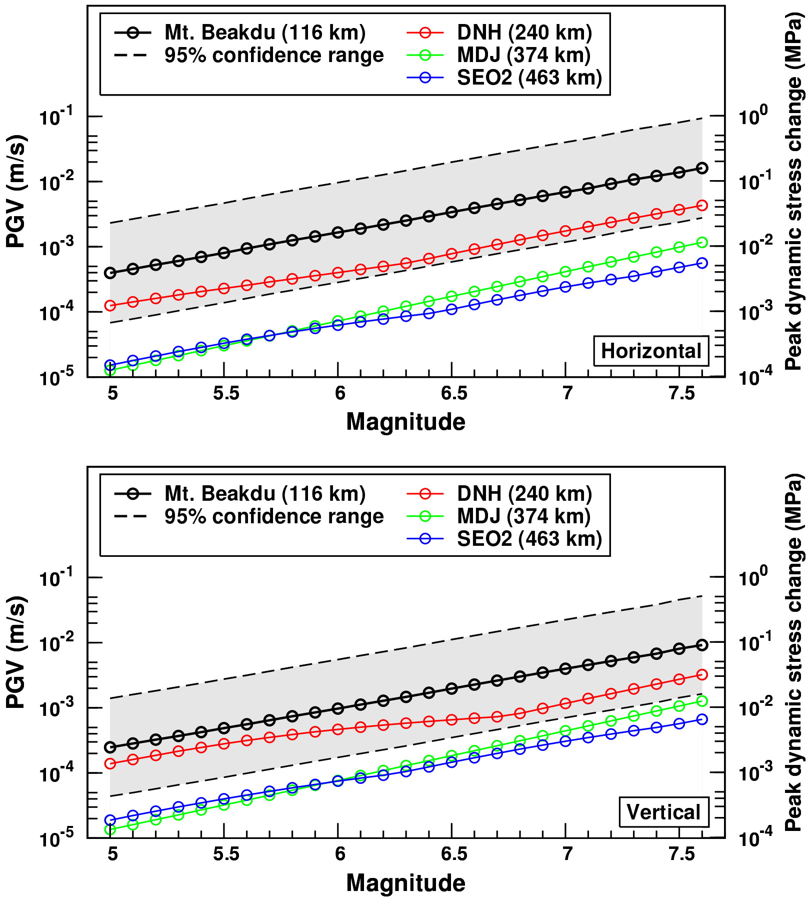

Figure 11. Variations of horizontal and vertical PGVs and equivalent peak dynamic stress changes as a function of magnitude on the ground of Baekdu volcano. The 95 % confidence ranges are marked in shade of gray.

Jolivet, L., K. Tamaki, and M. Fournier (1994). Japan Sea, opening history and mechanism: A synthesis, Journal of Geophysical Research, 99, 22237-22259.

Marin, S., J.-P. Avouac, M. Nicolas, and A. Schlupp (2004). A probabilistic approach to seismic hazard in metropolitan France, Bulletin of the Seismological Society of America, 94, 2137-2163.

Mueller, C. S. (1985). Source pulse enhancement by deconvolution of an empirical Green's function, Geophysical Research Letters, 12, 33-36.

Oh, C.W. (2006). A new concept on tectonic correlation between Korea, China and Japan: Histories from the late Proterozoic to Cretaceous, Gondwana Research, 9, 47-61.

Sereno, T. J., S.R. Bratt, and T.C. Bache (1988). Simultaneous inversion of regional wave spectra for attenuation and seismic moment in Scandinavia. Journal of Geophysical Research 93, 2019-2035.

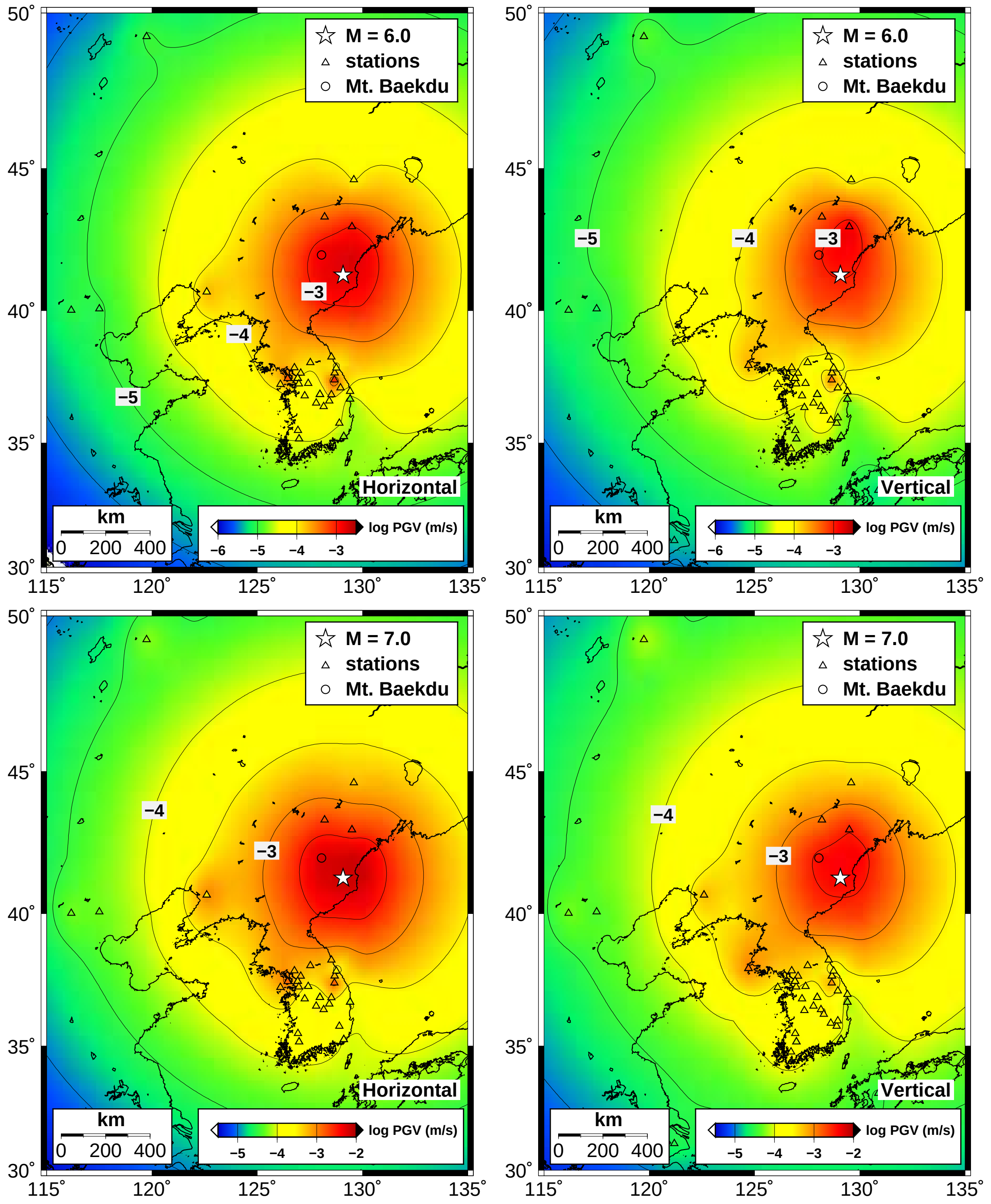

Figure 12. Spatial distribution of horizontal and vertical PGVs induced by UNEs of m_b 6.0 and 7.0. The maps were created using the software Generic Mapping Tools (http://gmt.soest.hawaii.edu/).

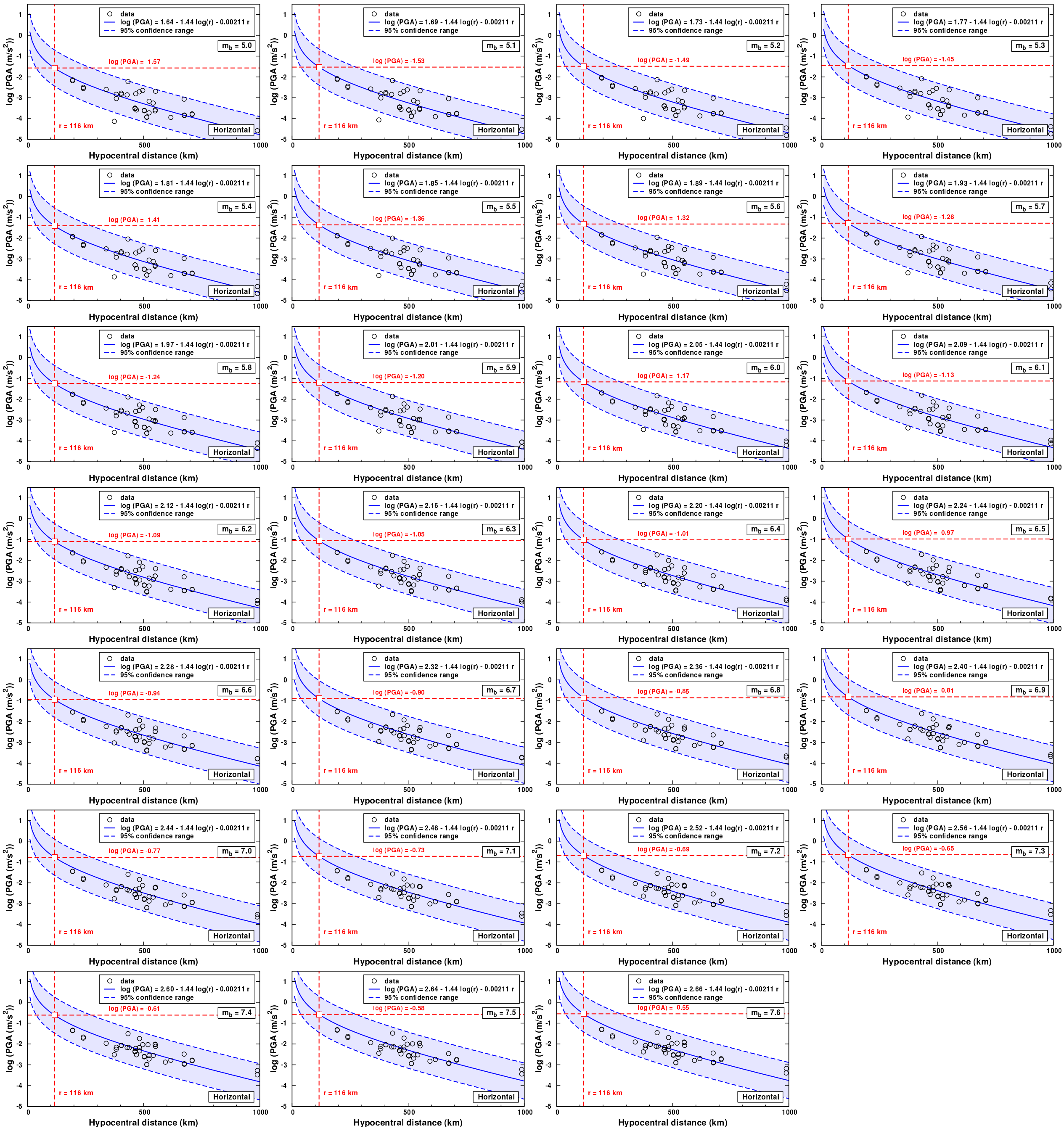

Figure 13. Determination of horizontal PGAs on the ground of Baekdu volcano for UNEs of m_b 5.0-7.6. The horizontal PGA attenuation curves are presented in solid blue lines with 95 % confidence ranges in shade of blue.

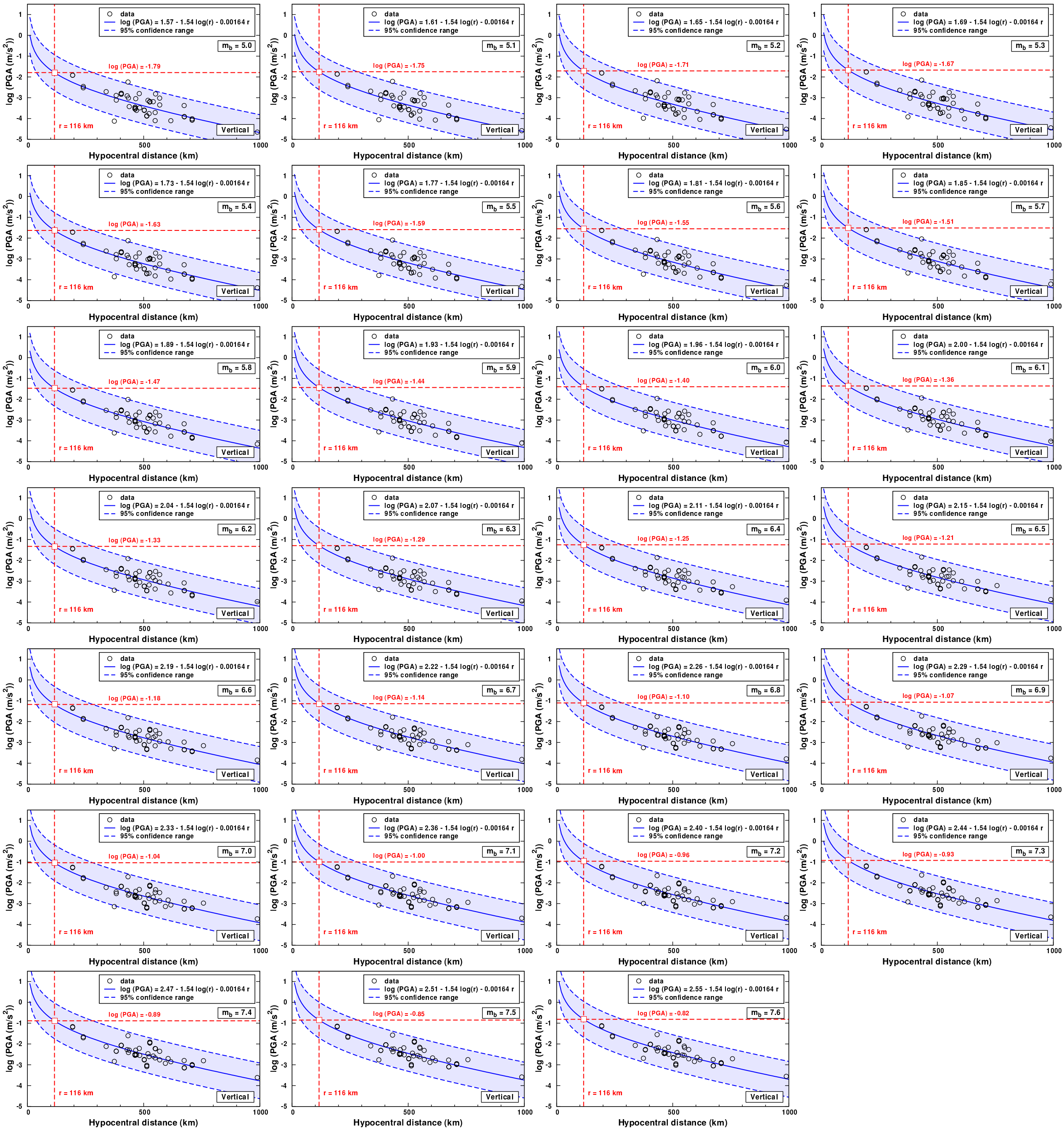

Figure 14. Determination of vertical PGAs on the ground of Baekdu volcano for UNEs of m_b 5.0-7.6. The vertical PGA attenuation curves are presented in solid blue lines with 95 % confidence ranges in shade of blue.

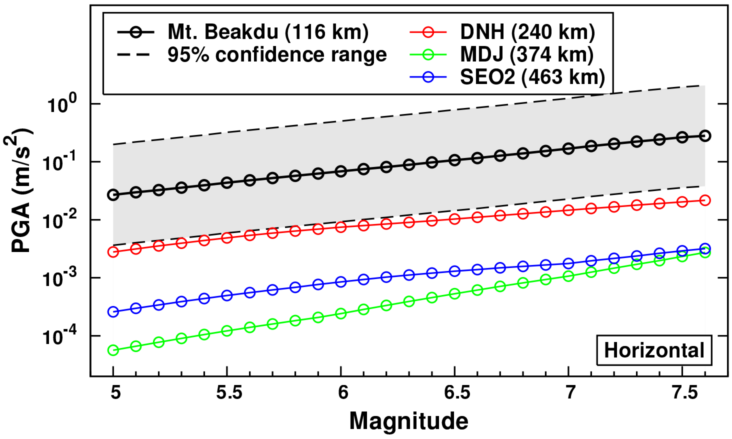

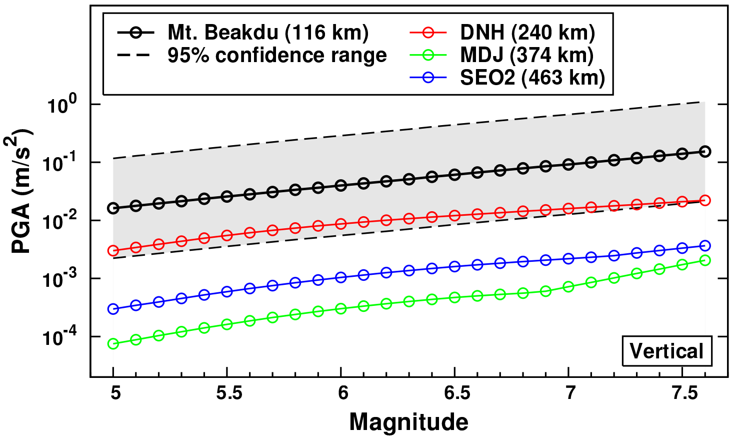

Figure 15. Variations of horizontal and vertical PGAs as a function of magnitude on the ground of Baekdu volcano. The 95 % confidence ranges are marked in shade of gray.

Shin, J.S., D.-H. Sheen, and G. Kim (2010). Regional observations of the second North Korean nuclear test on 2009 May 25, Geophysical Journal International, 180, 243-250.

Xie, J., and H.J. Patton (1999). Regional phase excitation and propagation in the Lop Nor region of central Asia and implications for P/Lg discriminants, Journal of Geophysical Research, 104 (B1), 941-954.

Zhang, M., and L. Wen (2013). High-precision location and yield of North Koreas 2013 nuclear test, Geophysical Research Letters, 40, 2941-2946, doi:10.1002/grl.50607.

Zhao, L.-F., X.B. Xie, W.-M. Wang, and Z.-X. Yao (2012). Yield estimation of the 25 May 2009 North Korean nuclear explosion, Bulletin of the Seismological Society of America, 102 (2), 467-478.

Zhao, L.-F., X.B. Xie, W.-M. Wang, and Z.-X. Yao (2014). The 12 February 2013 North Korean underground nuclear test, Seismological Research Letters, 85 (1), 130-134.

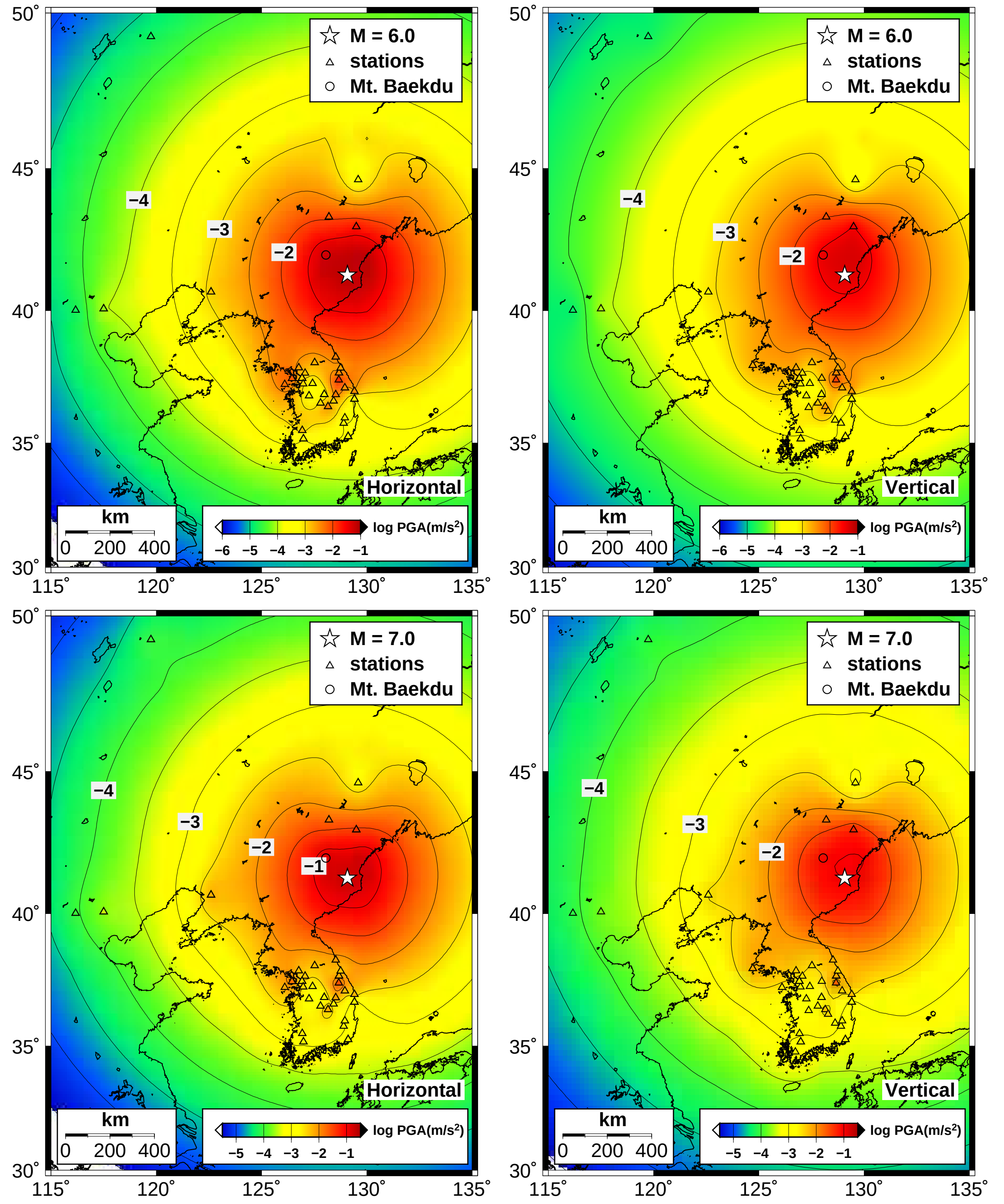

Figure 16. Spatial distribution of horizontal and vertical PGAs induced by UNEs of m_b 6.0 and 7.0. The maps were created using the software Generic Mapping Tools (http://gmt.soest.hawaii.edu/).
